# Extracellular matrix mediates moruloid-blastuloid morphodynamics in malignant ovarian spheroids

**DOI:** 10.26508/lsa.202000942

**Published:** 2021-08-10

**Authors:** Jimpi Langthasa, Purba Sarkar, Shruthi Narayanan, Rahul Bhagat, Annapurna Vadaparty, Ramray Bhat

**Affiliations:** 1 Department of Molecular Reproduction Development and Genetics, Indian Institute of Science, Bengaluru, India; 2 Sri Shankara Cancer Hospital and Research Centre, Bangalore, India

## Abstract

Ovarian cancer metastasizes into peritoneum through dissemination of transformed epithelia as multicellular spheroids. Harvested from the malignant ascites of patients, spheroids exhibit startling features of organization typical to homeostatic glandular tissues: lumen surrounded by smoothly contoured and adhered epithelia. Herein, we demonstrate that cells of specific ovarian cancer lines in suspension, aggregate into dysmorphic solid “moruloid” clusters that permit intercellular movement, cell penetration, and interspheroidal coalescence. Moruloid clusters subsequently mature into “blastuloid” spheroids with smooth contours, a temporally dynamic lumen and immotile cells. Blastuloid spheroids neither coalesce nor allow cell penetration. Ultrastructural examination reveals a basement membrane-like extracellular matrix coat on the surface of blastuloid, but not moruloid, spheroids. Quantitative proteomics reveals down-regulation in ECM protein Fibronectin-1 associated with the moruloid-blastuloid transition; immunocytochemistry also confirms the relocalization of basement membrane ECM proteins: collagen IV and laminin to the surface of blastuloid spheroids. Fibronectin depletion accelerates, and enzymatic basement membrane debridement impairs, lumen formation, respectively. The regulation by ECM dynamics of the morphogenesis of cancer spheroids potentially influences the progression of the disease.

## Abstract (precis)

Expression dynamics of basement membrane-like extracellular matrix and fibronectin regulate transitions between solid (moruloid) and cavitational (blastuloid) phenotypes of ovarian cancer spheroids.

## Introduction

Survival of women afflicted with epithelial ovarian cancer (EOC) trails behind other gynecological malignancies, despite improvements in surgical-pharmacological approaches (Vaughan et al, 2011; Siegel et al, 2015). The morbidity associated with the disease is a consequence of its transcoelomic route of metastasis: transformed epithelia of the fallopian tubes and ovaries in the form of spheroids (Moss et al, 2009; Lengyel, 2010), eventually home and adhere to the mesothelial lining of the peritoneum, occasionally invading through the underlying collagenous extracellular matrix to form secondary metastatic foci around abdominal organs (Burleson et al, 2004, 2006; Lengyel, 2010). EOC spheroids impede the drainage of the fluid from the peritoneal cavity and alter its composition; in turn, the fluid now known as malignant ascites serves as a pro-tumorigenic milieu for the spheroids (Latifi et al, 2012; Kipps et al, 2013).

The formation and presence of spheroids within ascites of ovarian cancer patients is strongly associated with recurrence of cancer and greater resistance to chemotherapy (Lee et al, 2013). Therefore, to develop novel strategies to target the spheroidal metastatic niche, it is essential to investigate mechanisms that underlie their morphogenesis. Several proteins have been proposed to mediate the adhesion between ovarian cancer epithelia that give rise to spheroids. These include transmembrane receptors such as CD44 (Sacks Suarez et al, 2019), cell adhesion molecules such as E-cadherin and N-cadherin (Klymenko et al, 2017), and matrix adhesion-inducing proteins such as integrins (Casey & Skubitz, 2000; Shield et al, 2007). Remarkably, a phase-contrast microscopic examination of spheroids from patients, or from aggregated epithelia of immortalized cancer lines cultured on low attachment substrata, shows features of morphogenetic organization: presence of a central lumen, radially arranged apposed epithelia and compacted spheroidal surfaces. Such traits are cognate to organized morphogenesis within the glandular epithelial organs (Nelson et al, 2006; Bhat & Bissell, 2014), which are built through principles that include, but are not limited to, cell–cell and matrix adhesion (Newman & Bhat, 2009; Benítez et al, 2018). In fact, loss of tissue architecture seen in tumorigenesis is characterized by the disappearance of such morphogenetic traits (such as matrix adhesion and polarity) (Hanahan & Weinberg, 2000; Bissell and Hines, 2011).

In this article, we investigate how these traits are recapitulated in a fluid metastatic context. Using spheroids from patients with high grade serous adenocarcinoma and ovarian cancer cell lines, we show that the changes in ECM are responsible for the conversion of an aggregated solid multicellular cancer cluster into one with a temporally dynamic cavitation, which has significant implications for its ability to negotiate through the confines of the peritoneal cavity.

## Results

**Figure S1.**
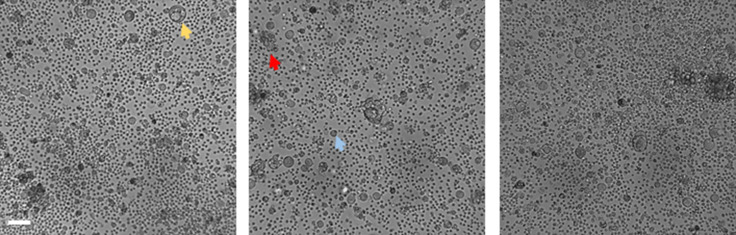
Bright-field images of patient spheroids showing lumen-less dysmorphic clusters (red arrowhead), lumen-containing spheroids (yellow arrowhead), and single cells (blue arrowhead) in a same sample. Scale bar: 50 μm (n ≥ 3).

The cellular fraction obtained from the malignant ascites of patients with EOCs (inclusion criteria: high grade serious ovarian carcinoma with symptom of ascites; exclusion criteria: mucinous or germ cell ovarian cancer, or cancer from other tissue origins with peritoneal metastases) was isolated and cultured on low attachment substrata. When observed under phase contrast microscopy, we found both single cells and multicellular aggregates within the cellular fractions (Fig S1). For most patients (>10 high grade serious ovarian carcinoma patients), aggregates with radially symmetric cellular architectures were observed. Such aggregates were mostly spherical in shape and are called spheroids: along with these dysmorphic non-spheroidal clusters were also visible.

**Figure 1.**
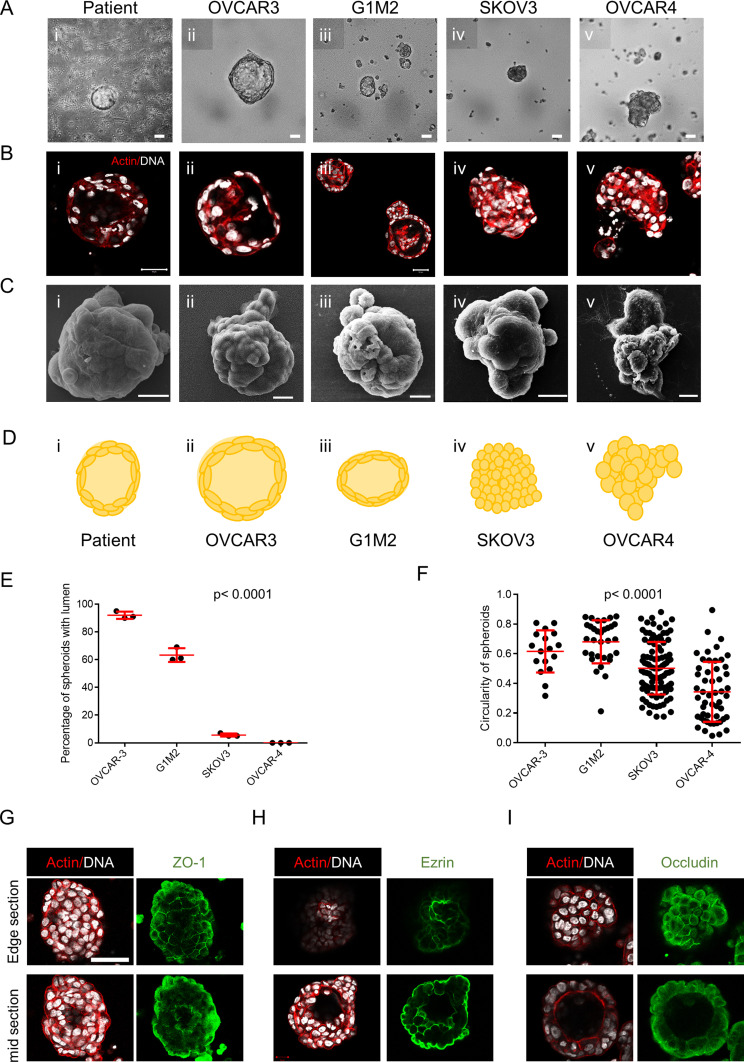
Spheroids of ovarian cancer epithelia show multicellular organization. **(A, B, C)** Photomicrographs of spheroids/clusters from patient malignant ascites (i), OVCAR3 (ii), G1M2 (iii), SKOV3 (iv), and OVCAR4 (v) cells imaged using bright-field microscopy (A), laser confocal microscopy staining DNA (DAPI; white) and F-actin (phalloidin; red) (B) and scanning electron microscopy (C) (n = 3, multiple spheroids analyzed for each repeat). **(D)** Cartoon depiction of the morphology of spheroids/clusters, highlighting lumen formation and outer contour based on (A, B, C). **(E)** Graph showing the percentage of lumen-containing spheroids from OVCAR3, G1M2, SKOV3, and OVCAR4 (n = 3, multiple spheroids analyzed for each repeat). Bars represent mean ± SD. Significance measured using one-way ANOVA. **(F)** Graph showing mean circularity of OVCAR3, G1M2, SKOV3, and OVCAR4 clusters from a representative experiment (n = 3, multiple spheroids analyzed for each repeat) Bars represent mean ± SD. Significance measured using one-way ANOVA. **(G, H, I)** Photomicrographs of OVCAR3 spheroids imaged using laser confocal photomicrography OVCAR3 spheroids stained for (G) ZO-1, (H) ezrin, and (I) occludin (all in green) and counterstaining for F-actin (red) and DNA (white) with the top row representing top edge z section of spheroids and bottom row representing mid Z section of the spheroids (n ≥ 2, multiple spheroids analyzed for each repeat). **(E, F)** Bars in (E, F) represent means ± SEM. **(A, B, C, G, H, I)** Scale bar for (A, B, G, H, I) is 50 μm and for (C) is 10 μm.

**Figure S2.**
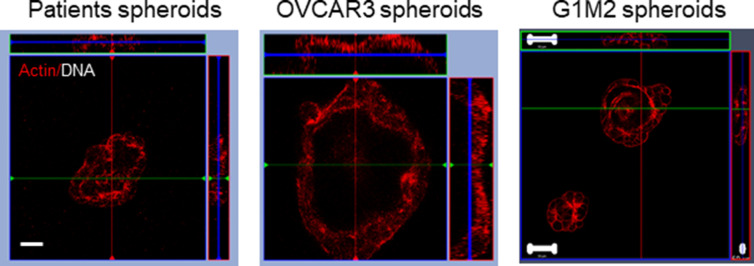
Laser confocal photomicrograph middle stack–orthogonal axes confirming the lumen present in the spheroids of patients (left) and OVCAR3 (middle) and G1M2 (right). Scale bar: 50 μm (n = 3).

**Figure S3.**
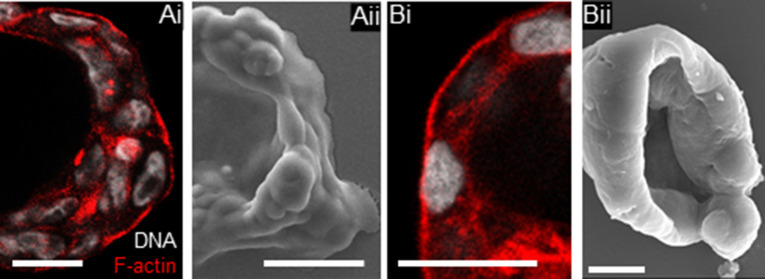
OVCAR3 and G1M2 blastuloid spheroids show similar cavitational morphologies. **(Ai, Aii, Bi, Bii)** A smooth cavitational contour seen for OVCAR3 spheroids based on laser confocal micrography (middle Z slice) (Ai) and scanning electron microscopy (Aii) as well as for G1M2 spheroids using laser confocal micrography (middle Z slice) (Bi), scanning electron microscopy (Bii). Scale bar for (Ai–ii) and (Bi): 50 μm, for (Bii): 10 μm (n = 3).

**Figure S4.**
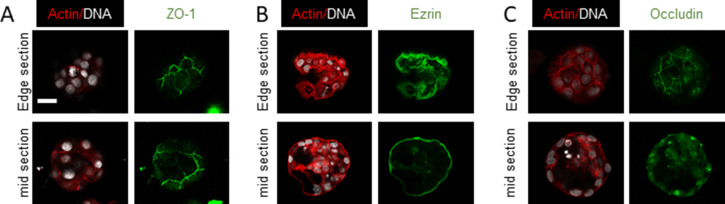
Staining of polarity markers (ZO-1, Ezrin and Occludin) in G1M2 blastuloid spheroids. **(A, B, C)** Photomicrographs of G1M2 blastuloid spheroids imaged using laser confocal photomicrography OVCAR3 spheroids stained for (A) ZO-1, (B) ezrin, and (C) occludin (all in green) and counterstaining for F-actin (red) and DNA (white) with the top row representing top edge z section of spheroids and bottom row representing mid Z section of the spheroids (n ≥ 2, multiple spheroids analyzed for each repeat). Scale bar: 50 μm.

We asked whether the dysmorphic and spheroidal morphologies represent distinct stages in a temporal spectrum of metastatic ovarian cancer morphologies. Testing the former hypothesis required an assay, wherein the formation of clusters from cells could be temporally tracked and quantifiably assessed. We cultured four ovarian cancer lines: OVCAR3, SKOV3, OVCAR4, and G1M2 (a xenograft-based cell line from an Indian patient with high grade serous ovarian adenocarcinoma [patient-derived xenograft]) on low-attachment substrata. The morphologies of their aggregates (along with ascites-derived spheroids) were observed over 7 d. The spheroids were fixed and stained with DAPI and phalloidin to visualize their nuclei and F-actin cytoskeleton, respectively, using bright-field or confocal microscopy (Fig 1A and B). Of the four cell lines, OVCAR3 and G1M2 consistently formed compacted spheroids with cavitations, SKOV3 formed spheroids that did not cavitate (although smaller hollow spheroids were observed) and OVCAR4 formed dysmorphic solid multicellular clusters. Digitally sectioning OVCAR3 and G1M2 clusters along three orthogonal planes with laser confocal microscopy confirmed the cavitation was indeed surrounded by cells (Fig S2). The surface of hollow spheroids examined by scanning electron microscopy (SEM) was smooth and compacted (Fig 1C). A cartoon depiction of the multicellular morphologies of spheroids from ascites and cell lines is shown in Fig 1D. Cell lines which gave rise to hollow clusters (Fig 1E) also showed a greater tendency for spheroidal morphology (Fig 1F). A closer examination of the inner lining of OVCAR3 and G1M2 spheroids revealed tightly apposed cancer epithelia forming a smooth boundary of the cavitation, suggesting the latter could be a result of cellular organization that involved establishment of intercellular junctions (Fig S3). To confirm this, we stained fixed spheroids for polarity markers ZO-1, ezrin, and occludin: we found strong outwardly radial intercellular localization for all three proteins on the outer surface of OVCAR3 and G1M2 spheroids as well as the luminal surface of OVCAR3 spheroids (Figs 1G–I and S4A–C). The predominantly outwardly radial staining of apical polarity proteins is also seen in vertebrate blastulation (Eckert & Fleming, 2008).

**Figure S5.**
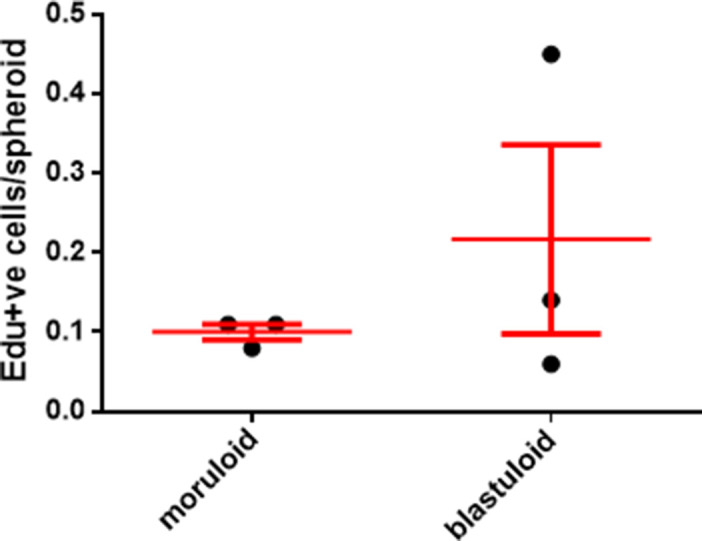
Graph showing the proportion of cells per spheroid that are staining for Edu compared between moruloid and blastuloid morphologies. Bars represent means ± SEM. Significance was measured using unpaired *t* test with Welch’s correction (n = 3).

**Figure 2.**
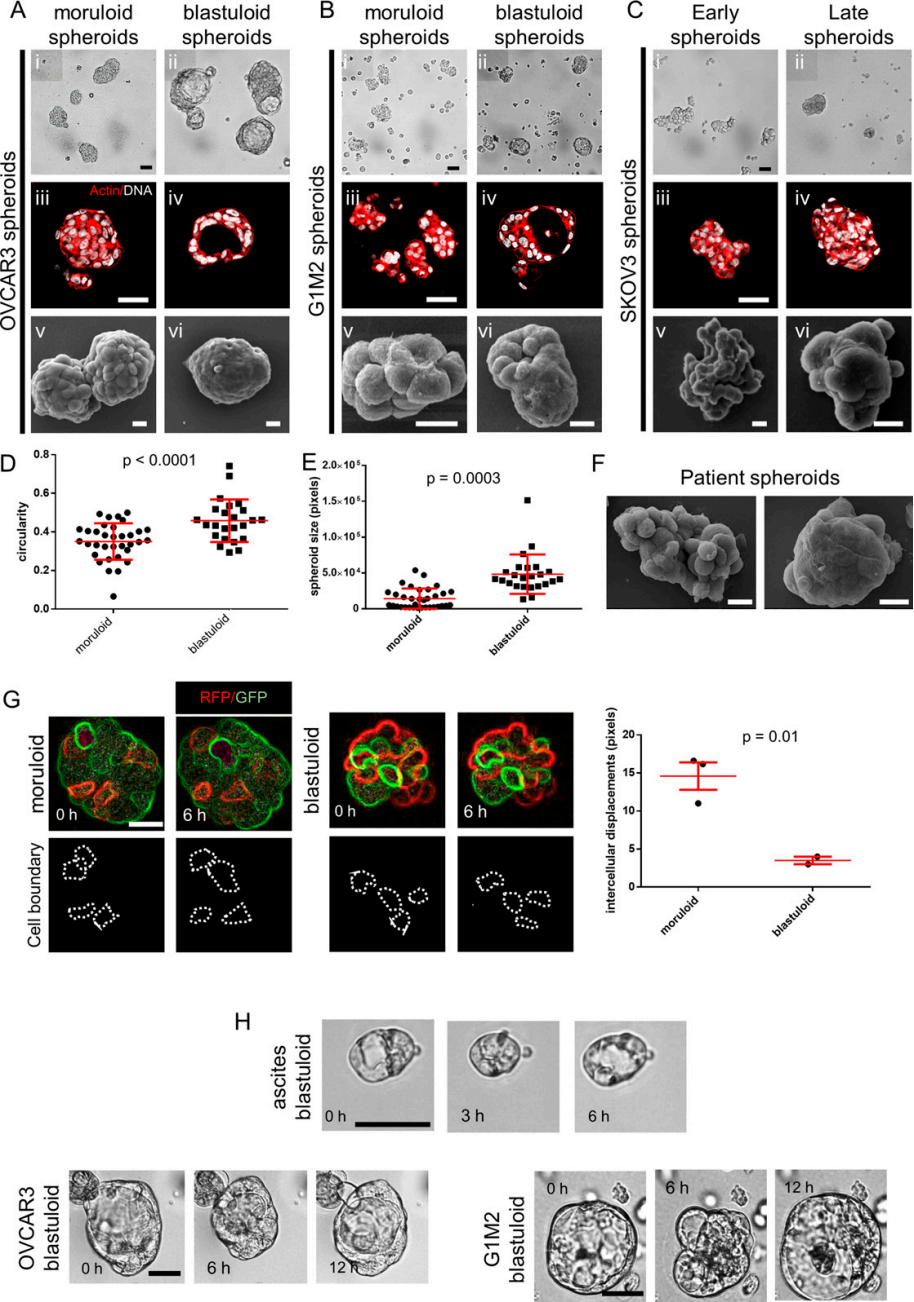
Distinctive features of moruloid and blastuloid morphologies in ovarian cancer spheroids. **(A, B, C)** Photomicrographs imaged using bright-field (i: moruloid, ii: blastuloid), laser confocal (iii: moruloid, iv: blastuloid; staining for DNA using DAPI [white] and for F-actin using phalloidin [red]), and scanning electron-microscopy (v: moruloid, vi: blastuloid) of OVCAR3 (A), G1M2 (B) and SKOV3 (C) spheroids (n = 3, multiple spheroids analyzed for each repeat). **(D, E)** Graphs showing change in size (D) and circularity (E) of OVCAR3 spheroids with moruloid (left) and blastuloid (right) morphologies from a representative morphometric experiment (n = 3 multiple spheroids analyzed for each repeat). Bars represent mean ± SD. Significance was tested using unpaired *t* test with Welch’s correction. **(F)** SEM photomicrographs of patient-derived spheroids showing moruloid (left) and blastuloid (right) morphologies (n ≥ 3, multiple spheroids analyzed for each repeat). **(G)** Photomicrographs taken at 0 and 6 h from time-lapse laser confocal videography of moruloid and blastuloid spheroids (constituted from a suspension of GFP- and red fluorescent protein (RFP)-expressing OVCAR3 cells) showing rearrangement of motile cells within them (white dotted lines highlight the position of motile cells) (edges rendered for fluorescence using Image J to show cell boundaries; see Videos S1 and S2). Graph on the right shows the difference in intercellular distance per unit time during time lapse videography (n = 3 multiple spheroids analyzed for each repeat). Bars represent mean ± SEM. Significance was tested using unpaired *t* test with Welch’s correction. **(H)** Bright-field micrographs of representative blastuloid spheroids from patient ascites (top), OVCAR3 (bottom left) and G1M2 (bottom right) imaged at regular time intervals showing temporal fluctuations in lumen size; see Videos S3–S5. (n = 3 independent repeats with multiple spheroids observed within the repeats). **(D, E, G)** Error bars in (D, E, G) signify median with interquartile range. Significance was measured using unpaired *t* test with Welch’s correction. **(A, B, Ci–iv, v–vi, F)** Scale bar for (A, B, Ci–iv) is 50 μm and for (A, B, Cv–vi) and (F) is 10 μm.

**Figure S6.**
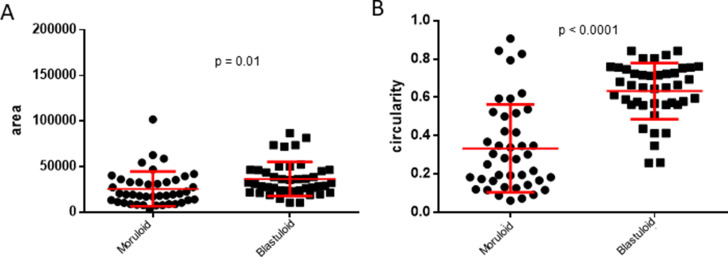
G1M2 blastuloid spheroids are bigger and more circular than moruloid spheroids. **(A, B)** Graphs showing change in size (A) and circularity (B) of G1M2 spheroids with moruloid (left) and blastuloid (right) morphologies in a representative morphometric experiment. Error bars signify mean ± SD. Significance was measured using unpaired *t* test with Welch’s correction (n = 3 independent experiments with multiple spheroids studied in each experiment).

**Figure S7.**
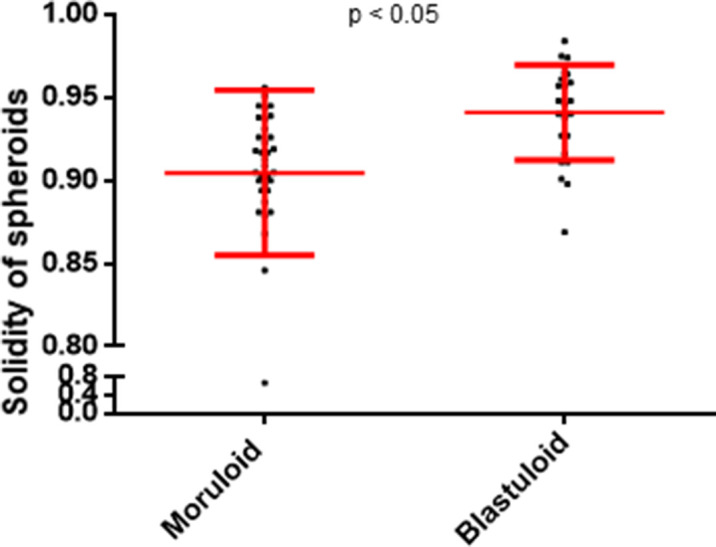
Graph showing change in solidity of OVCAR3 spheroids with moruloid (left) and blastuloid (right) morphologies. Error bars signify mean ± SD from a representative morphometric experiment. Significance was measured using unpaired *t* test with Welch’s correction (n = 2 independent experiments with multiple spheroids analyzed).

Within cell lines wherein we observed lumen formation, the process of spheroid formation could be divided into an initial early step of cellular aggregation, followed by cavitation over a more protracted culture period. The morphology of early OVCAR3 and G1M2 spheroids (cultured for 1–2 d) assumed a more “moruloid” appearance (no lumen, grape-like contour with cellular protuberances), whereas mature spheroids (cultured for 7 d) had a “blastuloid” appearance with compacted surface and a lumen. The temporal distinction in these two morphologies was confirmed with bright-field and phase-contrast microscopy (Fig 2Ai–ii and Bi–ii), fluorescent labeling of DNA and F-actin (Fig 2Aiii–iv and Biii–iv), and scanning electron microscopy (SEM) (Fig 2Av–vi and Bv–vi) in OVCAR3, G1M2, and to an extent in SKOV3 cells (Fig 2Ci–vi). The transition from moruloid to blastuloid phenotype was accompanied by an increase in size (Fig S6A, interestingly, the phenotypes did not show any difference in cell proliferation [Fig S5]), circularity (Figs 2D and E and S6B), and solidity (Fig S7).

**Figure S8.**
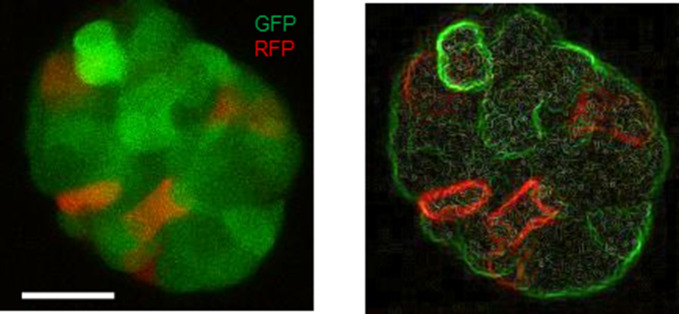
Rendering of GFP- and RFP-expressing cells through the “Find edges” plugin for Image J, wherein only cell edges are highlighted for better visualization of intercellular movement. Scale bar = 50 μm.

Our proposition of a two-step morphogenesis of spheroids also found support when analyzing ex vivo ascites cell fractions. Both moruloid and blastuloid spheroids were found within the cellular fractions obtained from malignant ascites of patients (Figs 2F and S1). The presence of lumen within homeostatic epithelial architectures is accompanied by emergence of coherent spatial relationships between neighboring cells because of establishment of intercellular junctions in coordination with cell polarity (Fournier et al, 2009; Fiore et al, 2017). We asked if the acquisition of blastuloid architecture is associated with such intercellular coherence through a decrease in intraspheroidal cell rearrangements. By videographing spheroids constituted through mixtures of OVCAR3 cells expressing GFP and RFP, we observed that moruloid spheroids were characterized by a dynamic rearrangement of motile cells within them (Fig 2G and Video S1; rendering of cell fluorescence edges for better imaging shown in Fig S8). On the other hand, such motility dynamics were not observed in case of blastuloid spheroids (Fig 2G and Video S2). To our surprise, our time lapse imaging also revealed the blastuloid spheroids to be dynamical structures with the lumen fluctuating in size and shape within patient ascites-derived spheroids and those from OVCAR3 and G1M2 cells (Fig 2H and Videos S3–S5).

Video 1 Moruloid OVCAR3 spheroid showing intercellular movement.Download video

Video 2 Blastuloid OVCAR3 spheroid showing no intercellular movement.Download video

Video 3 Ascitic blastuloid spheroids showing temporal fluctuations in lumen size.Download video

Video 4 OVCAR3 blastuloid spheroids showing temporal fluctuations in lumen size.Download video

Video 5 G1M2 blastuloid spheroids showing temporal fluctuations in lumen size.Download video

**Figure S9.**
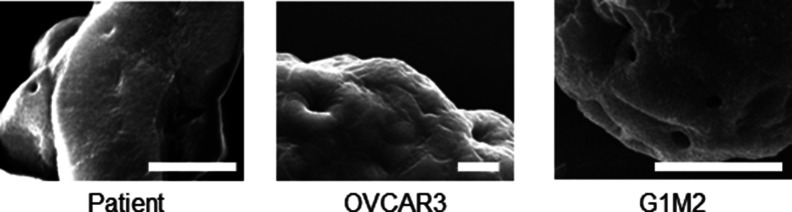
Scanning electron photomicrographs of patient, OVCAR3, and G1M2 spheroids showing a porous non-fibrillar ECM-like coating on the surface. Scale bar = 10 μm.

**Figure 3.**
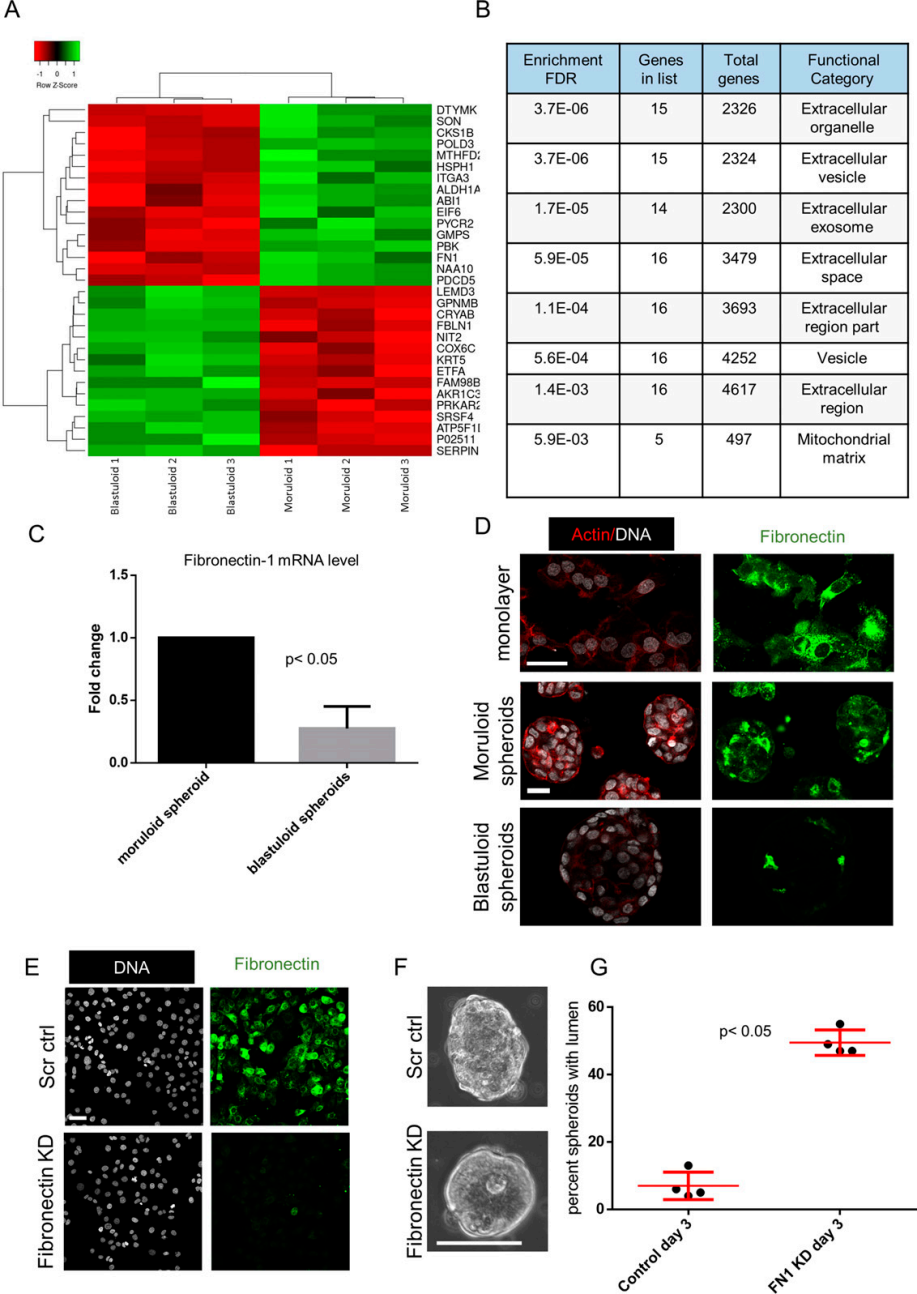
Moruloid and blastuloid OVCAR3 spheroids show distinct ECM expression dynamics. **(A)** Quantitative proteomic heat map clustered hierarchically between triplicate samples of OVCAR3 moruloid and blastuloid spheroids showing significantly up-regulated (green) and down-regulated (red) proteins. **(A, B)** Statistically significant enrichment of ontologies of the protein set shown in (A) based on cellular location (n = 3, cutoff: *P* < 0.005). **(C)** qPCR shows mRNA levels of Fibronectin-1 mRNA levels are decreased in blastuloid OVCAR3 spheroids compared with moruloid counterparts (*18sRNA* used as internal control; n = 3 independent biological experiments with at least duplicate samples run in each experiment). Error bars denote mean ± SEM. Paired *t* test was performed on ΔCt values for statistical significance (*P < 0.05). **(D)** Micrographs of OVCAR3 with laser confocal microscopy cultured as monolayers (top) moruloid spheroids (middle) and blastuloid spheroids (bottom) stained for Fibronectin-1 (green) and counterstained with F-actin (phalloidin; red) and DNA (DAPI; white) (n = 3). **(E)** Micrographs of OVCAR3 with laser confocal microscopy cultured as monolayers transduced with scrambled control shRNA (top) and shRNA against fibronectin (bottom) stained for Fibronectin (green) and counterstained with F-actin (phalloidin; red) and DNA (DAPI; white) (n = 3). **(F)** Phase contrast micrographs of representative OVCAR3 spheroids imaged at day 3 showing incipient lumen at day 3 upon fibronectin-1 knock down (bottom) compared with scrambled control (top) with a significant increase in lumen-containing spheroids in fibronectin-depleted spheroids **(G)** (n = 4, bars denote mean ± SEM). Significance tested using unpaired *t* test with Welch’s correction. Scale bar: 50 μm.

**Figure S10.**
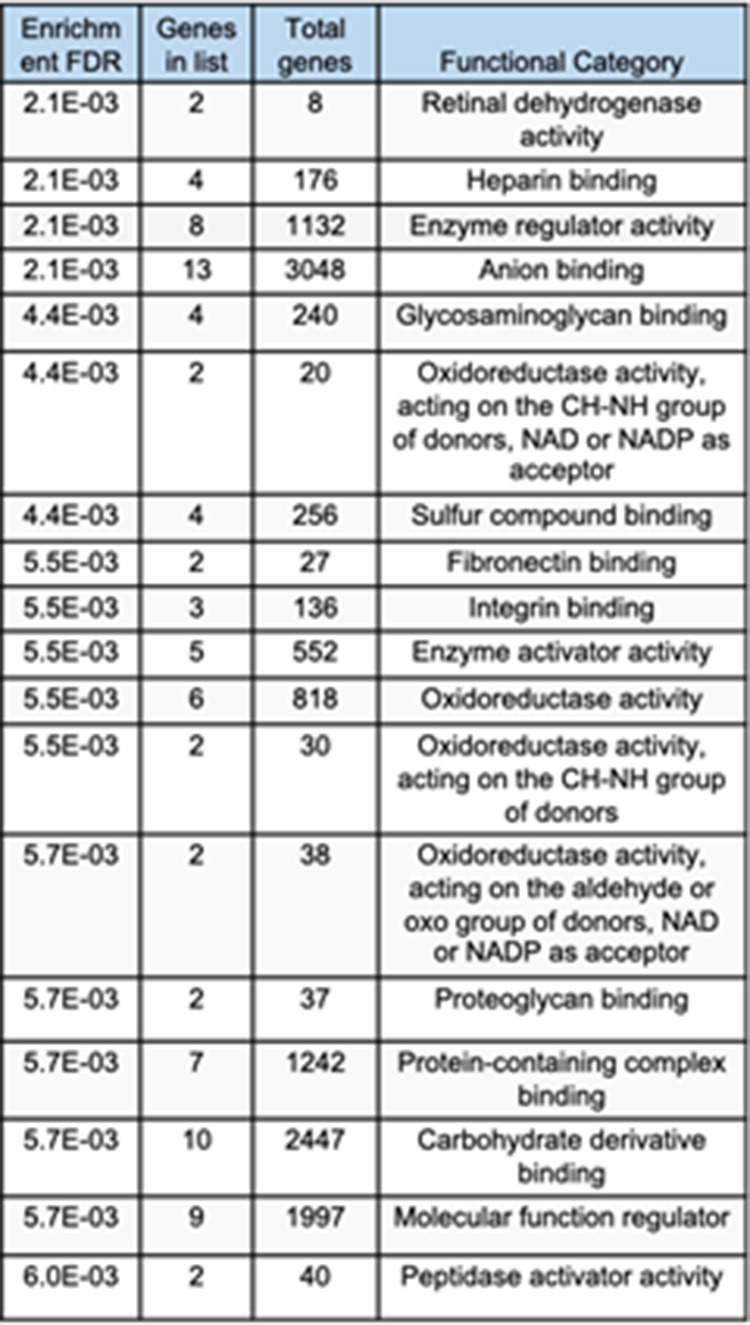
Statistically significant enrichment of ontologies of the protein set shown in Fig 3A (moruloid versus blastuloid spheroids) based on molecular function (n = 3, cutoff: P < 0.005).

**Figure S11.**
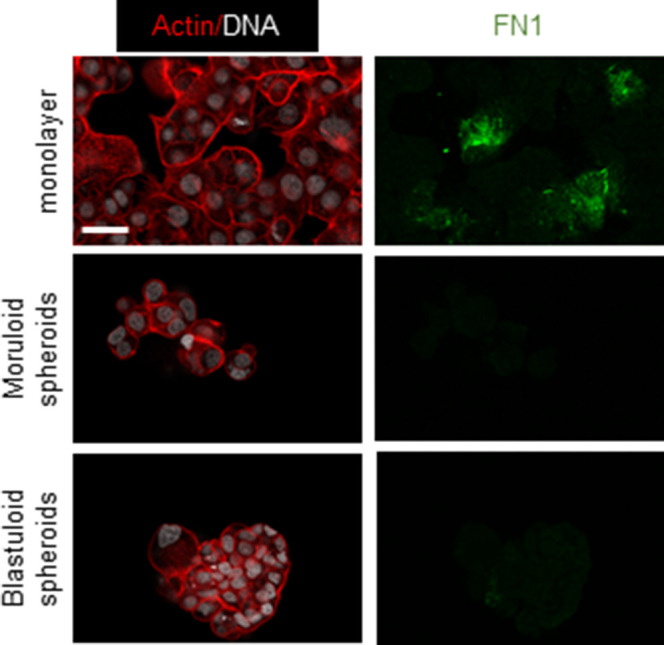
Micrographs of G1M2 with laser confocal microscopy cultured as monolayers (top), moruloid spheroids (middle) and blastuloid spheroids (bottom) stained for Fibronectin (green) and counterstained with F-actin (phalloidin; red) and DNA (DAPI; white) (n = 1). Scale bar: 50 μm.

**Figure S12.**
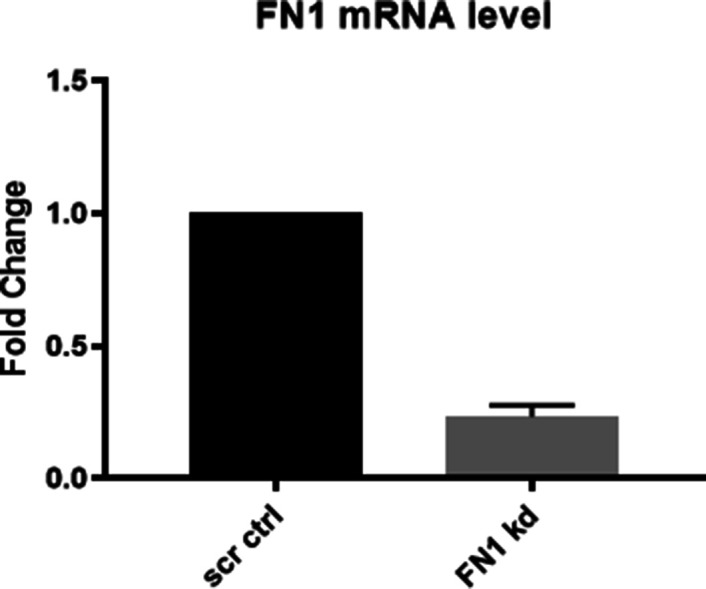
qPCR confirmation of *Fn1* knock down in OVCAR3 cell line. Showing decreased mRNA level in *Fn1* knock down compared to scrambled control (*18sRNA* used as internal control; n = 2 independent biological experiments with at least duplicate samples run in each experiment). Error bars denote mean ± SEM.

Examining the surface of ultrastructurally imaged blastuloid spheroids suggested the presence of a coat-like biomaterial that masked the furrows between the cells; such furrows are otherwise visible in moruloid spheroids (Fig 2A and Bv and vi). The non-fibrillar nature of the coat along with the presence of occasional pores suggested to us that mature spheroids may specifically be covered by a basement membrane (BM)-like ECM coat on their outer surface (Howat et al, 2001, 2002) (Fig S9). We used a quantitative proteomic approach to identify proteins that are relatively enriched within lysates of moruloid and blastuloid OVCAR3 spheroids (Fig 3A). Hierarchical clustering of the proteomic results showed 15 up-regulated and 16 down-regulated proteins in blastuloid spheroids compared with moruloid spheroids. Of these, fibronectin-1 (FN) was a notable ECM protein to be specifically down-regulated, in association with moruloid-to-blastuloid spheroid transition. Ontological analyses also showed extracellular processes and ECM element binding to be among the significantly enriched subcellular locations and molecular functions for the proteins altered between the two progressive stages of spheroid morphodynamics (Figs 3B and S10). We confirmed the down-regulation of *Fn* mRNA levels in blastuloid spheroids compared with their moruloid counterparts using real time quantitative PCR (Fig 3C). FN levels of OVCAR3 and G1M2 blastuloid spheroids were found to be down-regulated compared with OVCAR3 moruloid spheroids and monolayer cultivated OVCAR3 and G1M2 cells (Figs 3D and S11). This led us to hypothesize whether a down-regulation in FN could induce the morphological transition. To test this, we down-regulated *Fn* expression in OVCAR3 cells using lentiviral gene-cognate shRNA transduction (depletion was confirmed using immunocytochemistry Fig 3E and real time quantitative PCR, Fig S12). Consistent with our hypothesis, *Fn*-depleted spheroids showed a faster transition to blastuloid morphologies: at days 3 and 4, lumen could clearly be observed in a greater proportion of *Fn*-depleted spheroids than in control counterparts (Fig 3F and G).

**Figure 4.**
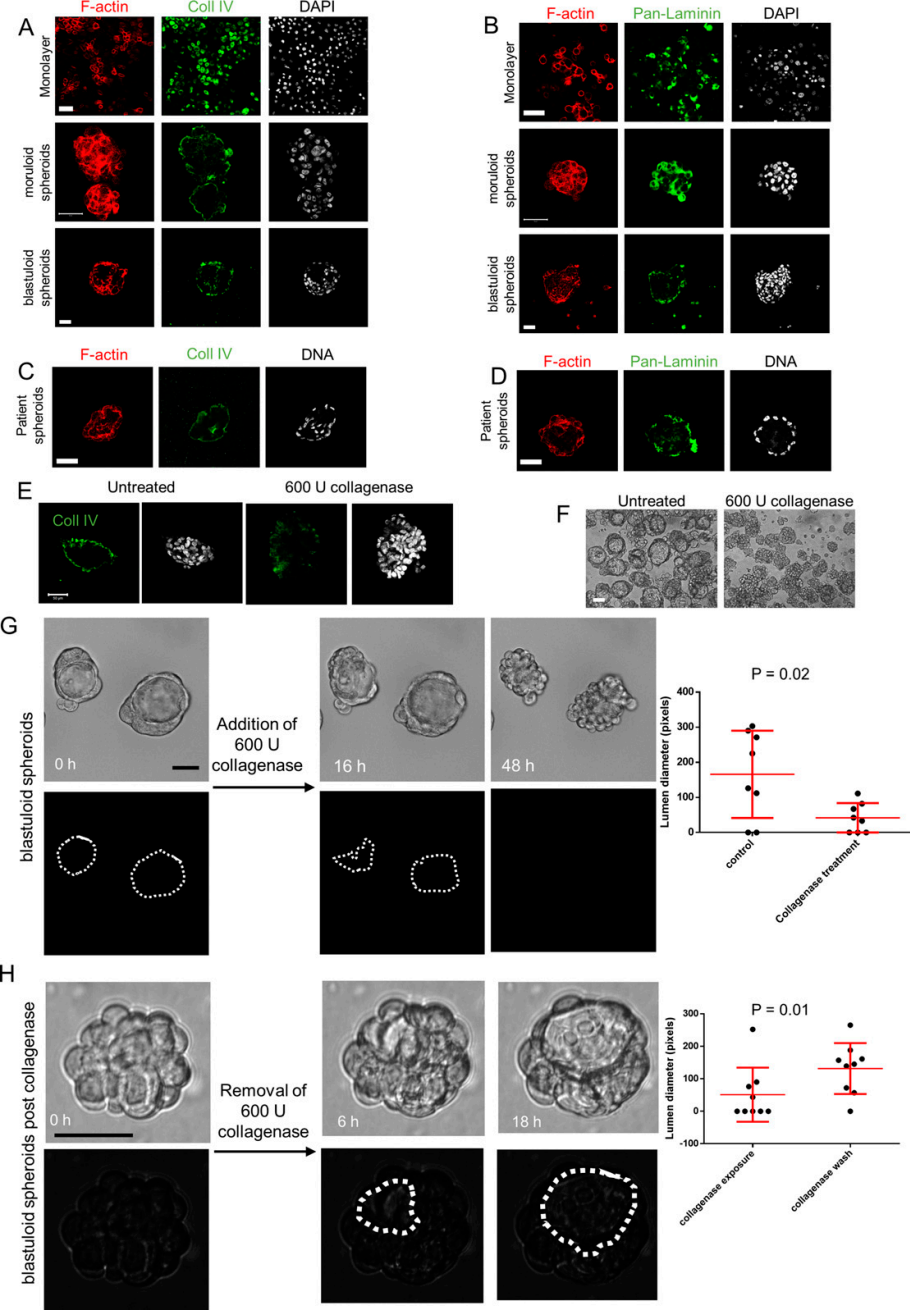
The presence of basement membrane-like ECM coat correlates with lumen formation in spheroids. **(A, B)** Laser confocal photomicrographs showing collagen IV (green) (A) and pan-laminin (green) (B) localization using indirect immunofluorescence in monolayers (top row) moruloid-(middle row) and blastuloid- (bottom row) spheroids from OVCAR3 cells counterstained for F-actin with phalloidin (red) and DNA with DAPI (white). **(C, D)** Laser confocal photomicrographs showing collagen IV (green) (C) and pan-laminin (green) (D) localization using indirect immunofluorescence in patient spheroids counterstained for F-actin with phalloidin (red) and DNA with DAPI (white). **(E)** Laser confocal photomicrographs stained for Collagen IV (green) and DNA (DAPI; white) in untreated control OVCAR3 spheroids (left) and upon treatment with Collagenase IV (right). **(F)** Phase-contrast photomicrographs showing the morphologies of spheroids with no treatment (control, left) and upon treatment with Collagenase IV (right). **(G)** Bright-field photomicrographs taken at 0, 16, and 48 h from time-lapse videography of blastuloid OVCAR3 spheroids initiated after addition of collagenase IV (see Video S6). **(G)** White dotted lines in the black background highlight the changes in the contour of lumen in (G). (three independent repeats with multiple spheroids analyzed for each repeat) Graph on the right shows change in lumen size calculated using paired *t* test. Bars represent mean ± SD from a representative experiment. **(H)** Bright-field photomicrographs taken at 0, 6,, and 18 h from time-lapse videography of OVCAR3 spheroids pretreated with Collagenase IV with videography initiated after the removal of Collagenase IV (see Video S7). **(H)** White dotted lines in the black background highlight the changes in the contour of lumen in (H). (n = 3 independent repeats with multiple spheroids analyzed for each repeat) Graph on the right shows change in lumen size calculated using paired *t* test. Bars represent mean ± SD from a representative experiment. **(A, B, C, D, E, F, G, H)** Scale bar for (A, B, C, D, E, F, G, H): 50 μm.

**Figure S13.**
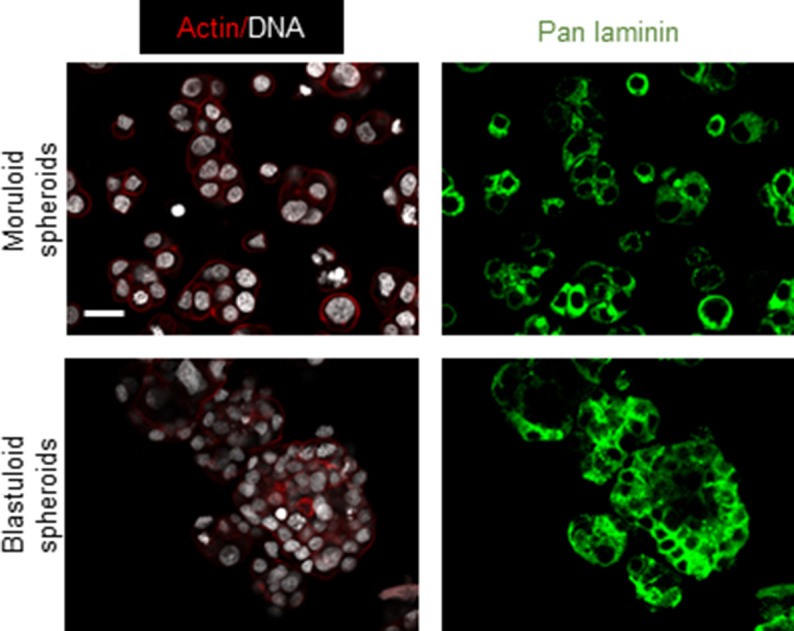
Laser confocal photomicrographs showing pan-laminin (green) localization using indirect immunofluorescence moruloid- (top row) and blastuloid- (bottom row) spheroids from G1M2 cells counterstained for F-actin with phalloidin (red) and DNA with DAPI (white) (n = 2). Scale bar: 20 μm.

**Figure S14.**
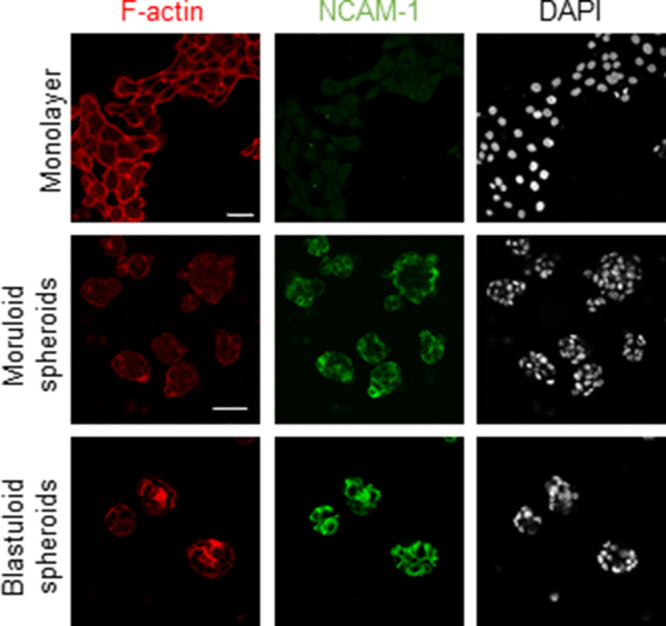
Laser confocal photomicrographs showing NCAM1 (green) localization using indirect immunofluorescence in monolayers (top row) moruloid-(middle row) and blastuloid- (bottom row) spheroids from OVCAR3 cells counterstained for F-actin with phalloidin (red) and DNA with DAPI (white) (n = 2). Scale bar: 50 μm.

Our observation of a BM-like coat in our microscopic analysis led us to cytochemically probe the specific canonical constituents of BM matrix. Non-fibrillar collagen IV and laminins typify the macromolecular organization of BM (Leblond & Inoue, 1989; Jayadev & Sherwood, 2017). We checked for the localization of these proteins in adhesive OVCAR3 monolayers and their moruloid and blastuloid spheroids. Collagen IV localized in the cytoplasm within monolayer OVCAR3 cells but was found to be present specifically in the outer surface of both moruloid and blastuloid spheroids (Fig 4A). Pan-laminin signals, on the other hand, while also observed in cytoplasm of monolayer and moruloid OVCAR3 cells, localized to the outer surface of blastuloid spheroids (Fig 4B) and to the outer layer of cells in G1M2 blastuloid spheroids (Fig S13). The early localization of Collagen IV on the outer surface of moruloid spheroids also suggests that the protein may have a key laminin-independent role in initiating BM formation during spheroidogenesis (this has recently been observed in *Caenorhabditis elegans* pharyngeal BM formation [Jayadev et al, 2019]). Staining with NCAM1 for blastuloid spheroids confirmed that antibodies could penetrate the inner cells within such specimen (Fig S14). We also found Collagen IV and pan-laminin signals on the outer surface of the patient-derived lumen containing spheroids (Fig 4C and D). Altogether, the formation of a BM-like ECM coat was coincident with the acquisition of blastuloid organization by mature spheroids.

**Figure S15.**
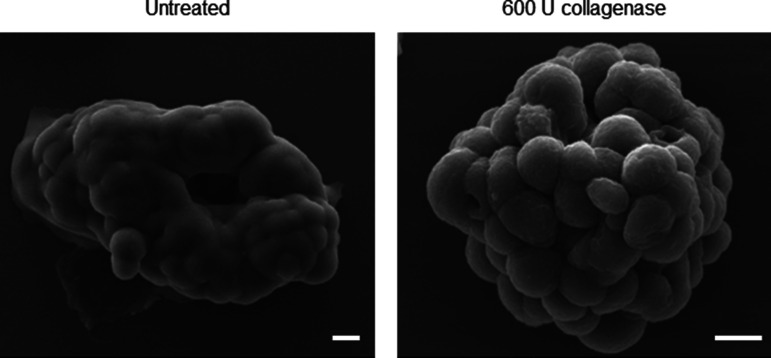
Scanning electron microscopy photomicrographs of blastuloid OVCAR3 spheroids untreated (left) and 600 U collagenase treated (right), confirming removal of ECM (n = 3). Scale bar = 10 μm.

**Figure S16.**
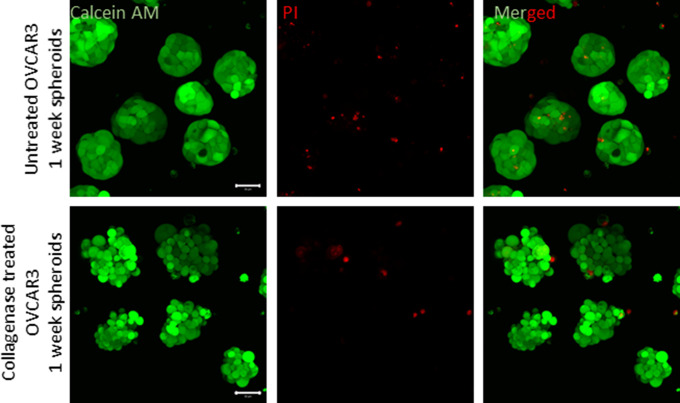
Micrographs of OVCAR3 blastuloid spheroids untreated (top) and collagenase treated (bottom) with laser confocal microscopy showing calcein AM-positive (green) and propidium iodide (red) (n = 2). Scale bar: 50 μm.

**Figure S17.**
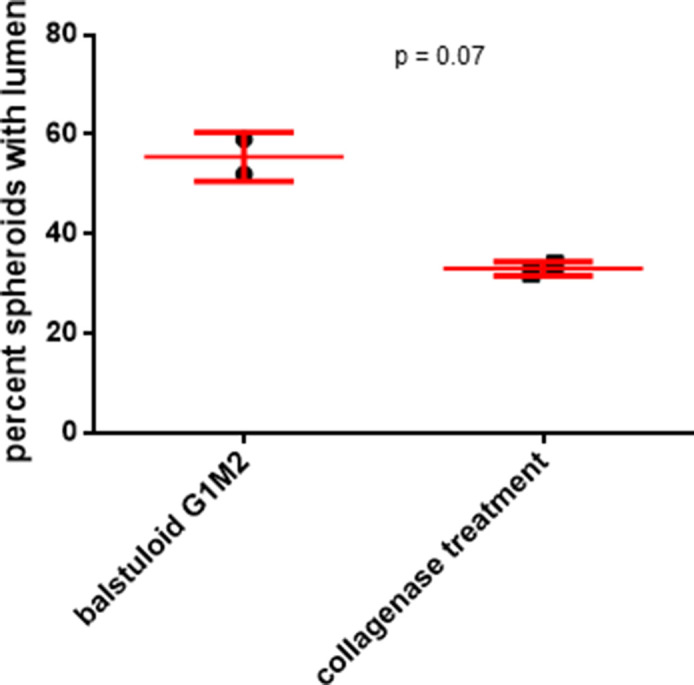
Graph showing relative percentage of lumen containing G1M2 blastuloid spheroids, untreated (left), and upon treatment with collagenase IV (right). Bars represent means ± SEM. Significance was measured using unpaired *t* test with Welch’s correction (n = 2).

To investigate whether the BM coat is involved in imparting stability to the morphology of the mature ovarian cancer spheroid, we treated them with collagenase IV (in presence and absence of its quencher, FBS). We confirmed the removal of matrix coat upon staining collagenase-treated spheroids for Collagen IV and evincing a decreased signal, when compared with control spheroids (Fig 4E). The removal of ECM coat was additionally confirmed using SEM, where we also noticed a reversal in compaction to a moruloid appearance with a grape-like contour (Fig S15; no discernible differences in viability were observed due to collagenase treatment [Fig S16]). Such changes in spheroidal morphology were also appreciable in phase contrast microscopy, wherein we detected an accompanying loss of lumen (Fig 4F; a depletion in lumen-associated morphology was also seen for G1M2 blastuloid spheroids Fig S17). We also tracked the process using time-lapse videomicrography, wherein a gradual and progressive depletion of lumen within spheroids (and a reversal of surface compaction) could be observed upon addition of collagenase (Fig 4G comparison between lumen diameter in control and collagenase-treated spheroids shown in graph on the right; dotted line marks the boundary of the lumen in Fig 4G and Video S6). Upon washing the collagenase away and re-culturing the spheroid in serum-free medium, we noticed a re-transition of the moruloid morphology to a blastuloid phenotype, with re-emergence of lumen and compaction of the surface within 24 h (Fig 4H; comparison between lumen diameter of spheroids in presence or washed depletion of collagenase shown in graph on the right; dotted line marks the boundary of the lumen in Fig 4H and Video S7). In fact, intercellular rearrangement and cell motility seen only in moruloid spheroids, also re-emerged in blastuloid spheroids upon debridement of BM by collagenase (Video S7). Our findings suggest that the BM coat mediates the transition of spheroids from an early moruloid- to a blastuloid-phenotype in a highly dynamic and reversible manner.

Video 6 Progressive loss of lumen and compaction in blastuloid spheroids upon exposure to collagenase IV.Download video

Video 7 Restoration in lumen and compaction in collagenase IV-treated blastuloid spheroids upon washing away the enzyme.Download video

**Figure 5.**
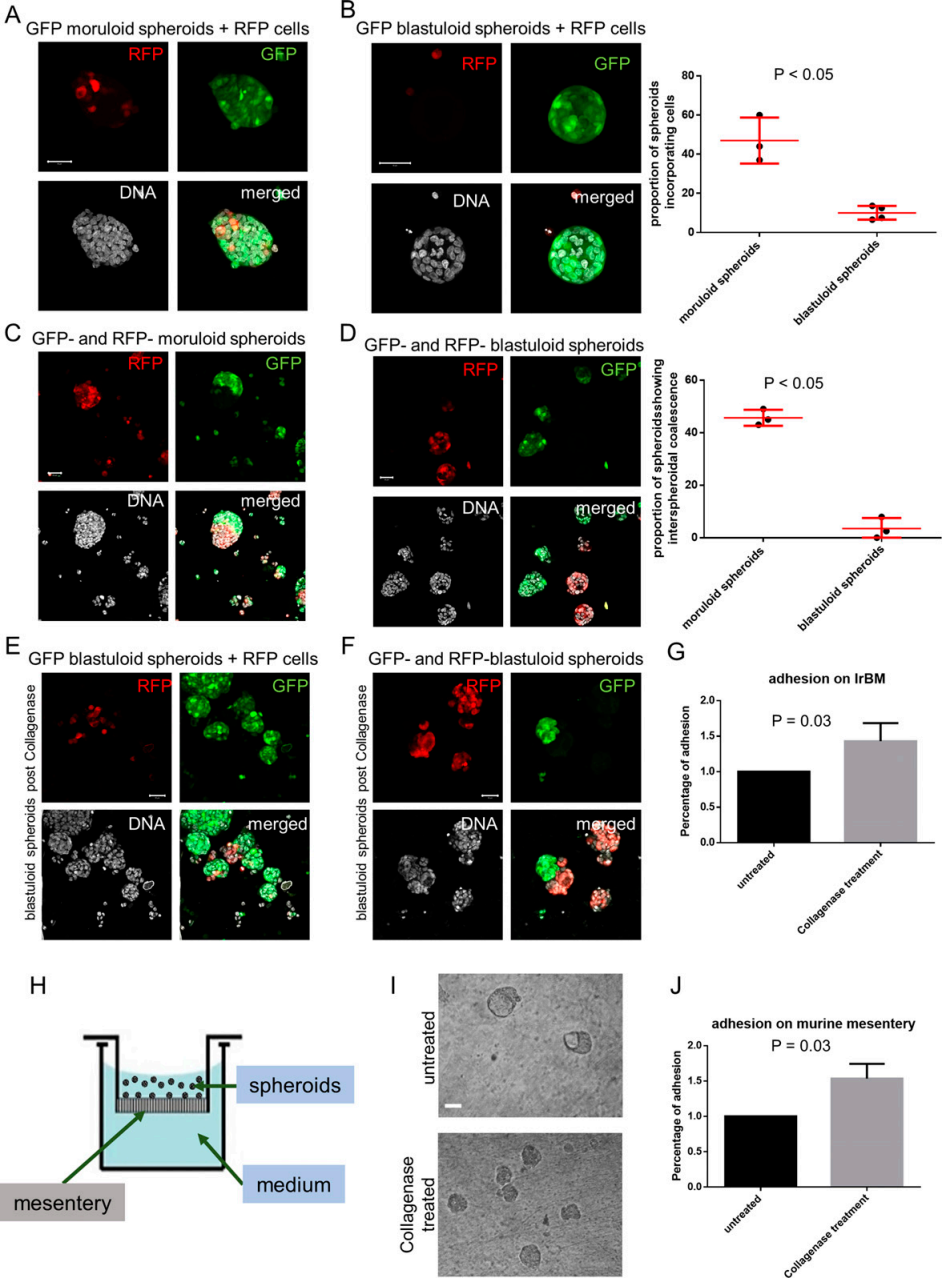
Blastuloid spheroids are morphogenetically more stable than moruloid spheroids. **(A, B)** Laser confocal photomicrographs of moruloid (A) and blastuloid (B) spheroids expressing GFP, which were cultured with single cells expressing RFP for 24 h and counterstained for DNA (DAPI; white) (n = 3 independent repeats with multiple spheroids analyzed for each repeat). Graph on the right shows differences in proportion of spheroids incorporating cells calculated using unpaired *t* test with Welch’s correction. Bars represent mean ± SD. **(C, D)** Laser confocal photomicrographs of spheroids initially formed from separate suspensions of GFP- and RFP-expressing OVCAR3 cells and then cultured together for 24 h and counterstained for DNA (DAPI; white) (n = 3 independent repeats with multiple spheroids analyzed for each repeat). Graph on the right shows differences in proportion of spheroids exhibiting coalescence calculated using unpaired *t* test with Welch’s correction. Bars represent mean ± SD. **(E)** Laser confocal photomicrographs of blastuloid spheroids expressing GFP, pretreated with collagenase IV and then cultured with single OVCAR3 cells expressing RFP for 24 h and counterstained for DNA (DAPI; white) (n = 3). **(F)** Laser confocal photomicrographs of blastuloid spheroids expressing GFP, pretreated with Collagenase IV and then cultured with blastuloid spheroids expressing RFP (also pretreated with Collagenase IV for 24 h and counterstained for DNA) (DAPI; white) (n = 3). **(G, H, I, J)** Bar graphs showing relative adhesion of blastuloid spheroids, untreated, and pretreated with collagenase IV, when cultured on top of laminin-rich basement membrane scaffolds (G), and 4–6-wk BALB/c murine mesenteries that are placed as substrata using transwells (schematic, H). Phase contrast micrographs of control (top) and collagenase-treated spheroids adhered to murine mesentery shown in (I) and proportion of adhesion shown in (J) (n = 3 independent repeats with multiple spheroids analyzed for each repeat). Bars represent means ± SEM. Significance was measured using ratio paired *t* test. **(A, B, C, D, E, F)** Scale bar for (A, B, C, D, E, F) 50 μm.

**Figure S18.**
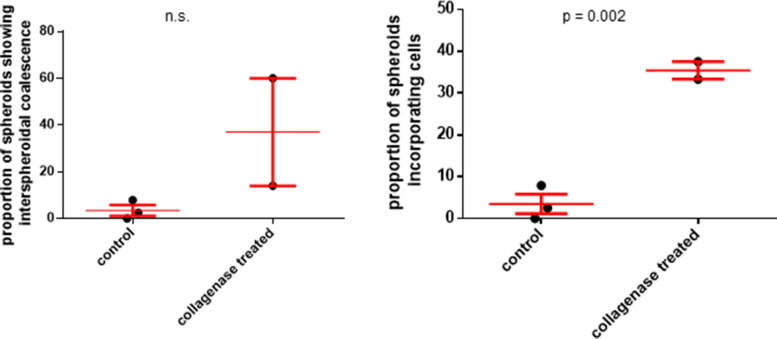
Graphs showing the proportion of blastuloid spheroids that show interspheroidal coalescence (left) or single cell incorporation (right) into their morphology with or without collagenase treatment. Bars represent means ± SEM. Significance was measured using unpaired *t* test (n = 3).

We next asked if the BM coat also regulates the size and cellular constitution of spheroids. To answer this question, we stably expressed GFP and RFP in separate populations of OVCAR3 cells. In the first experiment, we separately cultured moruloid and blastuloid GFP-expressing spheroids in suspension in the presence of RFP-expressing single OVCAR3 cells. Within 24 h, we observed RFP cells inside moruloid spheroids but not in blastuloid spheroids (Fig 5A and B; graph on the right showing statistically increased RFP cell incorporation within GFP spheroids). In the second experiment, we co-cultured, in suspension, moruloid GFP-expressing and RFP-expressing spheroids. Early spheroids were able to coalesce and give rise to bigger spheroids within 24 h of coculture (Fig 5C). When this same experiment was performed with mature spheroids, no coalescence was observed; mature spheroids remain sequestered without coalescing (Fig 5D; graph on the right showing significantly greater blastuloid spheroidal coalescence compared with moruloid morphologies). However, when the above experiment was repeated with blastuloid spheroids that had been treated with collagenase IV (and their BM coat debrided), there was a partial recapitulation of early spheroidal phenotype: we noticed the presence of RFP cells within BM-less GFP-expressing spheroids (Fig 5E). Coalescence was relatively uncommon although observed in some cases (Figs 5F and S18). Next, we asked whether, in addition to morphogenetic stability, the BM coat of spheroids also regulates their ability to attach to biological (cellular and matrix) substrata.

**Figure S19.**
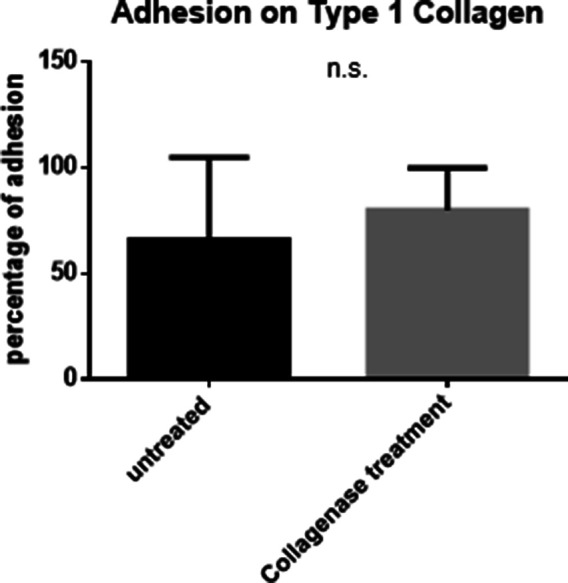
Bar graph showing relative adhesion of OVCAR3 blastuloid spheroids, untreated (left), and pretreated with Collagenase IV (right) when cultured on top of collagen I scaffold. Bars represent means ± SEM. Significance was measured using unpaired *t* test with Welch’s correction (n = 3).

We treated spheroids with collagenase IV and cultured them in suspension on top of laminin-rich BM (lrBM)- and type 1 collagen ECM (along with untreated controls). Interestingly, BM coat-debrided spheroids showed a significantly higher adhesion to lrBM matrices than their undebrided counterparts (Fig 5G). Higher adhesion was also observed for collagen I scaffolds although the mean percentages of adhesion were not significantly different from untreated controls (Fig S19). To recreate the prospective secondary metastatic microenvironment of ovarian cancer ex vivo, we dissected out the mesentery of 4–6-wk BALB/c mice and sewed it to the bottom of membrane-less transwells (Fig 5H). Upon adding the ovarian cancer spheroids (with and without BM), we assessed their adhesion to the mesothelial lining (visualization of spheroids, which retain their moruloid and blastuloid morphologies on murine mesenteries in Fig 5I). Spheroidal BM coat removal resulted in significantly better adhesion even on murine mesenteries when compared with untreated controls (Fig 5J).

## Discussion

**Figure S20.**
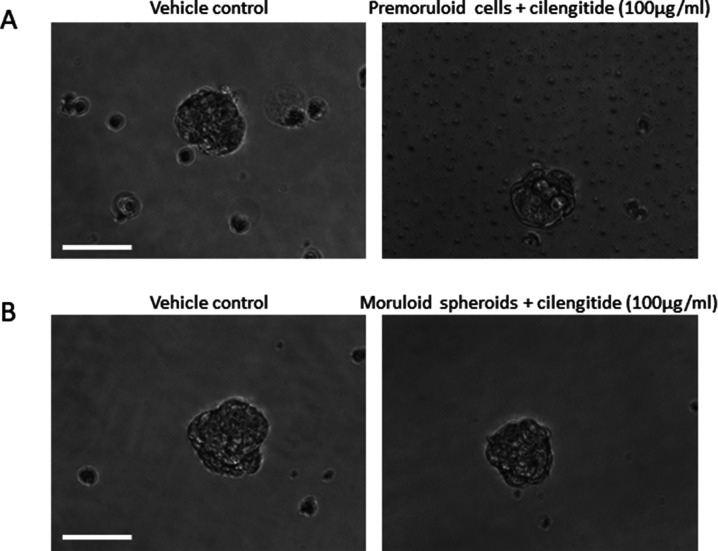
Phase contrast images of OVCAR3 spheroids which were treated with cilengitide (100 μg/ml) or vehicle control. **(A)** In the first experiment, cells were treated with vehicle (left) or cilengitide (right) before spheroidogenesis began. **(B)** In the second experiment, moruloid spheroids were treated with vehicle (left) and cilengitide (right) and observed after 3 d of culture (n = 1). Scale bar is 100 μm.

Tumors cells are known to secrete ECM that is proteomically and glycochemically distinct from that synthesized by epithelial and connective tissues (Socovich & Naba, 2019). In fact, the expression of ECM proteins and glycans by spheroids constituted from thyroid and glioma cancer cell lines has been demonstrated by previous studies (Nederman et al, 1984; Glimelius et al, 1988; Vallen et al, 2014). We extend these observations to demonstrate the importance of spatiotemporal expression of the BM-constituting proteins in the metastatic niche of ovarian cancer patients as well as cell lines. Furthermore, we demonstrate important functions mediated by this morphogenetic trait; the first relates to multicellular organization. The appearance of the BM results in loss in cell movement; compaction and stabilization of intercellular relationships and formation of lumen. In fact, down-regulation of fibronectin-1 in blastuloid spheroids, is consistent with its well-established role in driving migration of cancer cells (Graf et al, 2021). Moreover, fibronectin is a well-known mesenchymal marker, and its down-regulation in blastuloid spheroids, along with establishment of lumen, polarity, and a BM-like ECM coat implies a consonance between moruloid-to-blastuloid and mesenchymal to epithelial transition. Intriguingly, preliminary studies with cilengitide (a cyclic arginine-glycine-aspartate [RGD] pentapeptide and a known inhibitor of integrins, canonical ligands of fibronectin) had scant effect on spheroidal morphogenesis (Fig S20). Future efforts with integrin-specific inhibitors and wider concentration regimes will reveal if fibronectin regulates morphogenesis in an integrin-independent manner.

Cavitation within multicellular structures has been attributed to the apoptosis of centrally located cells in embryonic and postnatal contexts (Hoffman et al, 1996; Coucouvanis & Martin, 1999). In contrast, lumen formation may take place without apoptosis, through a separation of apical and basolateral organization within Marine Darby Canine Kidney cells cultured in collagen (Ojakian et al, 1997; O’Brien et al, 2010). Recent research on the formation of blastocoels within murine blastocysts attributes the latter to microfractures in cell–cell contacts followed by fusion of microlumina, similar to Ostwald ripening (Dumortier et al, 2019). The kinetics of lumen appearance and abrogation in ovarian cancer spheroids upon formation and removal of BM, respectively, suggests an apoptosis-independent mechanism. This is further strengthened by its concurrence with stoppage of intercellular rearrangements that may be detrimental to the establishment of cell–cell contacts necessary for both polarity- and microfracture-dependent mechanisms. Our observations on moruloid-to-blastuloid spheroidal transitions are better aligned with a putative mechanism of spheroid formation through intraperitoneal aggregation of disseminated ovarian cancer cells. However, they do not foreclose the possibility of spheroidogenesis through the exfoliation of multicellular sheets as has been proposed recently, especially if the latter program results in formation of hollow collectives through an intermediate non-cavitational phenotype. We aim to explore the morphological transitions of exfoliated multicellular morphologies in greater details in the future.

**Figure S21.**
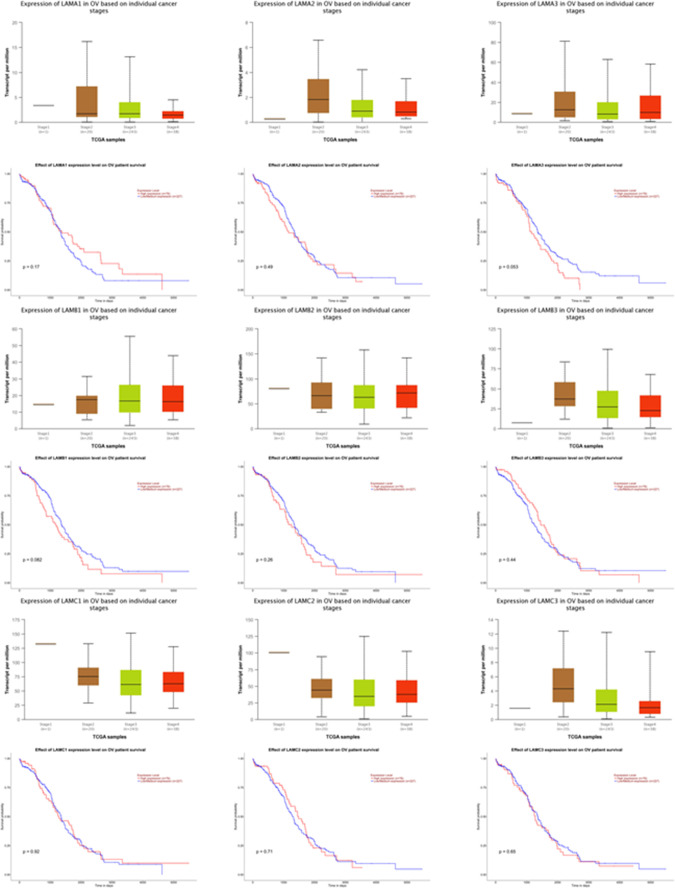
Stage-specific changes in expression of laminin encoding genes, as well as effects of their expression on overall survival in patients with cystadenocarcinoma curated within the cancer genome atlas database through UALCAN (Chandrashekar et al, 2017).

The literature on size regulation in spheroids is scarce. The self-limiting behavior of spheroids has been proposed to depend on proliferation or diffusion of nutrients (LaRue et al, 2004; Achilli et al, 2012). Besides proliferation, spheroids may also grow through interspheroidal coalescence (Susienka et al, 2016; Kim et al, 2018). Our observations show that the gradual formation of the BM may act as a barrier to coalescence and incorporation of new cells from the suspended milieu. In doing so, the BM formation also facilitates the spheroids to adopt a dual morphological phenotype with distinct rheological properties: coalescence of BM-coatless moruloid spheroids is prognostic of liquid-like behavior (Gonzalez-Rodriguez et al, 2012), whereas BM-containing blastuloid spheroids are relatively more solid-like with minimal compositional rearrangement. Such morphological dichotomy might significantly impact how spheroids traverse through peritoneal spaces during metastasis. Laminins represent one of the principal constituents of the BM ECM. The cancer genome atlas data suggest robust expression of several laminin-encoding genes in advanced stages of EOC, although the effect of their expression on overall survival is not clear with the exception of LAMA3 (Fig S21) (Chandrashekar et al, 2017). However, earlier studies have noted an ascites-specific enrichment of laminin in ovarian cancer patients (Byers et al, 1995); in addition, murine studies show an increased peritoneal survival of ovarian cancer cells in the presence of laminin (Yoshida et al, 2001). Our observations provide a morphological mechanism for the contributions of BM proteins in ovarian cancer progression.

The colonization of secondary metastatic sites has long been interpreted through the framework of the “seed-and-soil” hypothesis (Paget, 1889). Recent research furthers the hypothesis by proposing that premetastatic niche often carries with it agents such as activated fibroblasts (that can act as the soil) that facilitate the colonization (Duda et al, 2010). Our results indicate that the “soil” carried may be complex and may not necessarily be inducive: the BM coat of blastuloid spheroids actually impedes colonization relative to the moruloid ones. The consequences of such negative regulation could be linked to the ascitic accumulation of spheroidal population, which may induce collagenase secretion by peritoneal mesothelia (Mizutani et al, 2000). Our findings therefore suggest a link between the buildup of the ovarian cancer metastatic niche and its mesenteric colonization, rendering it an important target for future studies.

## Materials and Methods

### Cell culture

The human ovarian cancer cell lines: OVCAR3, OVCAR4, and SKOV3 were a kind gift from Professor Rajan R. Dighe, Indian Institute of Science. OVCAR3 and OVCAR4 were maintained in DMEM (AL007A; HiMedia) supplemented with 10–20% FBS (10270; Gibco) and antibiotics in a humidified atmosphere of 95% air and 5% CO2 at 37°C. The SKOV3 cell line was maintained in McCoy’s 5A medium (AL275A; HiMedia) supplemented with 10% FBS and antibiotics. The ovarian cancer cell line G1M2 (patient-derived xenograft line) was a kind gift from Professor Sharmila A. Bapat, the National Centre for Cell Science (NCCS), India. G1M2 cell line was maintained in Roswell Park Memorial Institute medium (AL162A; HiMedia) supplemented with 10% FBS and antibiotics.

### Spheroid culture

Spheroids were cultured in tissue culture dishes coated with 3% poly-2-hydroxyethyl methacrylate (polyHEMA) (P3932; Sigma-Aldrich) solution. Culture dishes were coated overnight under sterile conditions. PolyHEMA solution was prepared in 95% absolute ethanol. PolyHEMA coating prevented cell attachment and allowed spheroid formation in suspension. Cells were seeded according to the requirement for experiment in defined medium: DMEM: F12 (1:1) (HiMedia AT140) supplemented with 0.5 µg/ml hydrocortisone (Sigma-Aldrich, H0888), 250 ng/ml insulin (Sigma-Aldrich, I6634), 2.6 ng/ml sodium selenite (Sigma-Aldrich, S5261)), 27.3 pg/ml estradiol (Sigma-Aldrich, E2758), 5 µg/ml prolactin (Sigma-Aldrich L6520, 10 µg/ml transferrin (Sigma-Aldrich, T3309). Spheroids were visualized using phase contrast or bright-field microscopy and collected from the cultures by centrifugation at 0.1–0.2*g* for 5 min.

### Clinical samples

Ascites obtained from the peritoneal tap of patients with ovarian cancer was provided by Sri Sankara Cancer Hospital with due ethical clearance. Patient spheroids were cultured in tissue culture-treated polystyrene substrata/polyHEMA coated dish using DMEM (AL007A; HiMedia)—supplemented with 10–20% FBS (10270; Gibco) and antibiotics or with defined medium. Spheroids were then collected from the cultures by centrifugation at 0.1–0.2*g* for 5 min.

### Immunostaining and image acquisition

Cells were fixed using 3.7% formaldehyde (24005; Thermo Fisher Scientific) at 4°C for 20 min. After fixation, the cells were taken for further processing or stored in 1× PBS at 4°C. For monolayer culture, cells were directly seeded in an eight well chambered cover glass for immunostaining, whereas spheroids were first pelleted in a sterile 15-ml falcon tube followed by fixing, washing, and resuspension in PBS. Following this, 10–20 μl of spheroid suspension was put in an eight well chambered cover glass and dried by placing on a dry bath at 37°C for 15–30 min. Permeabilization was achieved using 0.5% Triton X-100 (MB031; HiMedia) for 1–2 h at RT. Effective permeabilization is needed for entry and uniform exposure to the antibodies. Blocking was achieved using PBS with 0.1% Triton X-100 and BSA (MB083; HiMedia) for 45 min at RT. Primary antibody incubation was carried out overnight at 4°C. This was followed by washes using 0.1% TritonX-100 in PBS (5 min × 3). Secondary antibody incubation was performed at RT for 2 h under dark conditions. DAPI (D1306; Thermo Fisher Scientific) was added to the samples and washed away after 15 min. Subsequent processing was carried out in the dark. This included washes using 0.1% Triton X-100 in PBS (5 min × 3). Images were captured in 20× using a Carl Zeiss LSM880 laser confocal microscope. Images were processed and analyzed using ZEN Lite software. The antibodies used in our studies are against ZO-1 (ab96587), ezrin (PA5-82769), occludin (71–1,500), FN1 (E5H6X), collagen IV (ABIN2889913), pan-laminin (ab11575), and NCAM-1 (ab9018). The antibody against ezrin was a kind gift of Prof. Shagufta Parveen, School of Regenerative Medicine, Manipal Academy of Higher Education. Negative controls in each case were through omission of the primary antibody.

### Genetic perturbation of fibronectin-1 (*Fn1*) gene

The *Fn1* gene shRNA clone was obtained from the MISSION shRNA library (Sigma Merck). Plasmid containing shRNA or scrambled control was packaged into lentiviruses using packaging vectors pMD2.G and psPAX2 (packaging vectors were a kind gift from Professor Deepak K Saini, MRDG, Indian Institute of Science). The plasmids were transfected into 293FT cells (R70007; Thermo Fisher Scientific) using TurboFect (R0533; Thermo Fisher Scientific). 293FT cells were cultured in DMEM supplemented with 10% FBS; conditioned medium containing viral particles was collected at 48 and 72 h. After filtering through a 0.45 μm filter, viral particles were concentrated using the Lenti-X concentrator according to the manufacturer’s protocol (631232; TaKaRa). Concentrated virus was aliquoted and stored at −80°C until use. OVCAR3 cells were seeded in a 24-well plate at 50−60% confluence and transduced with viral particles containing shRNA or scrambled control along with polybrene (8 μg/ml) for 24 h. After 72 h, transduced cells were selected using 5 μg/ml puromycin (CMS8861; HiMedia). The knock down of the gene was confirmed using real-time qPCR and immunofluorescence.

### Removal of spheroidal matrix using collagenase

Spheroids were cultured in 35 mm dishes by seeding 1.5 × 10^5^ cells for 1 wk. Collagenolysis of mature spheroids was performed using 600 U of collagenase IV (C5138; Sigma-Aldrich) in defined medium for 24 h. FBS was used for quenching the collagenolytic activity. Collagen-debrided spheroids were then fixed and processed for immunocytochemistry or scanning electron microscopy, using untreated 1 wk spheroids as control. Later, to check the effect of collagen debridement on spheroidogenesis, time lapse imaging was performed with blastuloid spheroids in the presence of collagenase using a bright-field epifluorescence microscope (IX73; Olympus). A detailed protocol of the time lapse microscopy is given below.

### SEM

Cellular monolayers and spheroids were fixed using 2.5% glutaraldehyde (0875; Amresco) overnight followed by three PBS (5 min each) washes to remove excess fixative. This was followed by five washes with water to remove salts. Dehydration was followed using different grades of ethanol (30%, 50%, 70%, 90%, and 100%). Spheroids were then put on 1 N HCl-treated coverslips and allowed to air dry completely at RT. Cellular monolayers were directly seeded on top of the treated coverslip. Imaging was performed using ESEM Quanta.

### Adhesion assay on murine mesenteries

BALB/c female mice (4–6 wk old) were used for adhesion experiments. Mice were euthanized by cervical dislocation and upon surgically dissecting the abdomen, their mesentery were strung to the lower ends of transwell inserts (Boyden chambers without the membranes) using surgical thread. The transwells containing mesentery were then placed in a sterile 24-well tissue culture plate and defined medium added into the transwell above and below the insert into wells of the plate. Spheroids were then added onto the upper layer of the mesentery in the insert; making sure the media does not spill from the insert into the well. Adhesion was studied using bright-field microscopy. Seeded spheroids were counted before wash (0 h) and after wash with PBS at 6 h time point.

### ECM coating for adhesion assay

Eight well chambered cover glasses (0030742036; Eppendorf) were coated with 50 μg/ml Growth factor-reduced BM matrix (Matrigel) (354230; Corning) or 1 mg/ml rat tail collagen I (A10483-01; Gibco) neutralized on ice in the presence of 10× DMEM with 0.1 N NaOH such that the final concentration of the collagen I is 1 mg/ml.

### Adhesion assay

Spheroids were cultured for 1 wk in polyHEMA-coated 35 mm dishes using defined medium. After 1 wk of culture, spheroids were treated with type IV collagenase (17104019; Gibco) for 24 h. Collagenase activity was quenched using 10% FBS, followed by PBS washes; untreated spheroids were used as control. Spheroids were resuspended in 1 ml defined medium for the assay. 10–20 μl of both untreated and treated spheroids suspension were put on top of ECM-coated chamber wells and isolated mesenteries in transwells. The number of spheroids seeded was counted, allowed to attach for 6 h by incubating them inside a humidified 37°C 5% CO_2_ incubator. After 6 h of incubation, the medium was replaced with fresh PBS to wash away unattached spheroids and the adhered ones were counted. The percentage of adhesion was calculated by dividing the number of spheroids attached to the substrata by the total number of spheroids seeded.

### Bright-field time lapse microscopy

Spheroids from cell lines were cultured for 24 h and 1 wk by seeding 1.5 × 10^5^ cells in a 35 mm cell culture dish. At particular time points, spheroids were harvested by centrifugation at 0.2*g* for 5 min, resuspended and put on a drop of 4% noble agar (A5431; Sigma-Aldrich) that was smeared on a glass-bottomed chamber well, which after some time was flooded with defined medium. Time-lapse imaging was subsequently performed for 48 h with 15 min interval using a Tokai Hit stage-top incubator with image acquisition through an Orca Flash LT plus camera (Hamamatsu) on an Olympus IX73 microscope.

### Confocal time lapse microscopy

We established GFP- and RFP-expressing OVCAR3 cell lines using lentiviral transduction. Using these cell lines, we cultured spheroids for 24 h or 1 wk on PolyHEMA-coated 35-mm dishes either individually or in combination, as described in the results section. Spheroids (made from mixtures of RFP- and GFP-expressing cells) were then harvested and immobilized for time lapse microscopy on a bed of 4% noble agar following the protocol described above. Time lapse imaging was performed using LEICA SP8 confocal microscope for 6 h with 15 min interval. Data were analyzed using LASX Leica software.

### Mass spectrometry

25 μl samples were taken and reduced with 5 mM tris(2-carboxyethyl) phosphine (TCEP) and further alkylated with 50 mM iodoacetamide and then digested with Trypsin (1:50, trypsin/lysate ratio) for 16 h at 37°C. Digests were cleaned using a C18 silica cartridge to remove the salt and dried using a speed vac. The dried pellet was resuspended in buffer A (5% acetonitrile, 0.1% formic acid). All the experiment was performed using EASY-nLC 1,000 system (Thermo Fisher Scientific) coupled to Thermo Fisher-QExactive equipped with nanoelectrospray ion source. 1.0 μg of the peptide mixture was resolved using 15 cm PicoFrit column (360 µm outer diameter, 75 µm inner diameter, 10 µm tip) filled with 2.0 μm of C18-resin (Dr Maeisch). The peptides were loaded with buffer A and eluted with a 0–40% gradient of buffer B (95% acetonitrile, 0.1% formic acid) at a flow rate of 300 nl/min for 100 min. MS data were acquired using a data-dependent top10 method dynamically choosing the most abundant precursor ions from the survey scan. All samples were processed, and RAW files generated were analyzed with Proteome Discoverer (v2.2) against the Uniprot HUMAN reference proteome database. For Sequest search, the precursor and fragment mass tolerances were set at 10 ppm and 0.5 D, respectively. The protease used to generate peptides, that is, enzyme specificity was set for trypsin/P (cleavage at the C terminus of “K/R”: unless followed by “P”) along with maximum missed cleavages value of two. Carbamidomethyl on cysteine as fixed modification and oxidation of methionine and N-terminal acetylation were considered as variable modifications for database search. Both peptide spectrum match and protein false discovery rate were set to 0.01 FDR. Statistical analysis was performed by using in-house R script. Abundance value for each run (including all biological replicates) were filtered and imputed by using normal distribution. Log2 transformed abundance values were normalized using Z-score. ANOVA and *t* test was performed based on *P*-value (threshold *P* < 0.05) to identify the significant proteins (Supplemental Data 1).

### Validation of differential gene expression by RT-qPCR

Quantitative real-time PCR was performed for *Fn1* gene where *18S rRNA* was used as internal control for the normalization of RT qPCR data. Total RNA was isolated using RNAiso Plus from OVCAR3 monolayer and spheroids, after which 1 μg of total RNA was reverse transcribed to cDNA using Verso cDNA Synthesis kit as per the manufacturer’s protocol (AB-1453; Thermo Fisher Scientific). Real-time qPCR was performed on Applied Biosystems 7500 Real-Time PCR System (Applied Biosystems) using a standard two-step amplification protocol followed by a melting curve analysis. The amplification reaction mixture (total volume of 10 μl) contained 10 ng of cDNA, 5 μl 2× DyNAmo Flash SYBER Green master mix, and 0.25 μM of the appropriate forward and reverse primer. Cycling condition: 95°C/10 min; 40 cycles of 95°C/15 s, annealing at 60°C/30 s for both the genes and extension at 72°C/15 s. Primer sequences of *Fn1* gene and *18S rRNA* gene, *Fn1* forward: CAAGCCAGATGTCAGAAGC, *Fn1* reverse: GGATGGTGCATCAATGGCA, *18 S* forward GTAACCCGTTGA ACCCCATT, *18 S* reverse CCATCCAATCGGTAGTAGCG. Relative gene expression was calculated using the comparative Ct method, and gene expression was normalized to moruloid spheroids. Appropriate no template and no-RT controls were included in each experiment. All the samples were analyzed in triplicates and repeated three times independently.

### Coculture experiments

GFP- and RFP-expressing OVCAR3 cell lines were used to investigate the stability of spheroids’ morphology. GFP-expressing OVCAR3 spheroids were cultured for 24 h or 1 wk in PolyHEMA-coated 35-mm dishes. To it were added RFP-expressing single cells or RFP-expressing spheroids cultured for 24 h and 1 wk time point, respectively, which then cocultured for 24 h. After fixation and stained with DAPI, spheroids were imaged by confocal microscopy.

### Statistical analysis

All experiments were performed in duplicates or more. All experiments were repeated thrice independently. Prism software (GraphPad Prism 6.0) was used for the generation of graphs and analysis. For all experiments, results are represented as mean ± SEM unless mentioned. For statistical analysis, an unpaired *t* test with Welch’s correction was performed in most cases unless appropriately specified in specific experiments. 

Supplemental Data 1. Raw values of results of proteomic comparison between moruloid and blastuloid OVCAR3 spheroids.

## Supplementary Material

Reviewer comments
